# Venereal and Urinary Diseases

**Published:** 1894-05

**Authors:** 


### BOOK NOTICES.

Venereal and Urinary Diseases. By Temple S. Hoyne, A. M., M. D., Clinical Professor of Skin and Venereal Diseases in Hering Medical College and Hospital, of Chicago. Second Edition, Revised and Enlarged. Halsey Bros., Chicago, Detroit, and Buffalo. John Morris Co., Printers, Chicago. 1894.

This volume of about 125 pages was originally prepared at the request of members of the College classes of 1881, 1882, and 1883. It consists almost entirely of the lectures delivered before the students. These lectures were arranged so as to give as brief and yet as comprehensive account of the subject as possible.

As a result, we have in the handy volume under review an admirable and withal interesting account of the whole subject. The homœopathic remedies are given with the specific indications. That these indications are reliable need hardly be said to any one who knows Dr. Hoyne as the editor of *The Medical Visitor*. More clear ideas can be gathered in a few minutes' perusal of this book than by any elaborate study of abstruse works on these subjects.

